# Homer1a signaling in the amygdala counteracts pain-related synaptic plasticity, mGluR1 function and pain behaviors

**DOI:** 10.1186/1744-8069-7-38

**Published:** 2011-05-19

**Authors:** Anke Tappe-Theodor, Yu Fu, Rohini Kuner, Volker Neugebauer

**Affiliations:** 1 Department of Neuroscience & Cell Biology, The University of Texas Medical Branch, 301 University Blvd., Galveston, Texas 77555-1069, USA; 2 Pharmakologisches Institut, Universität Heidelberg, Im Neuenheimer Feld 366, D-69120 Heidelberg, Germany

## Abstract

**Background:**

Group I metabotropic glutamate receptor (mGluR1/5) signaling is an important mechanism of pain-related plasticity in the amygdala that plays a key role in the emotional-affective dimension of pain. Homer1a, the short form of the Homer1 family of scaffolding proteins, disrupts the mGluR-signaling complex and negatively regulates nociceptive plasticity at spinal synapses. Using transgenic mice overexpressing Homer1a in the forebrain (H1a-mice), we analyzed synaptic plasticity, pain behavior and mGluR1 function in the basolateral amygdala (BLA) in a model of arthritis pain.

**Findings:**

In contrast to wild-type mice, H1a-mice mice did not develop increased pain behaviors (spinal reflexes and audible and ultrasonic vocalizations) after induction of arthritis in the knee joint. Whole-cell patch-clamp recordings in brain slices showed that excitatory synaptic transmission from the BLA to the central nucleus (CeA) did not change in arthritic H1a-mice but increased in arthritic wild-type mice. A selective mGluR1 antagonist (CPCCOEt) had no effect on enhanced synaptic transmission in slices from H1a-BLA mice with arthritis but inhibited transmission in wild-type mice with arthritis as in our previous studies in rats.

**Conclusions:**

The results show that Homer1a expressed in forebrain neurons, prevents the development of pain hypersensitivity in arthritis and disrupts pain-related plasticity at synapses in amygdaloid nuclei. Furthermore, Homer1a eliminates the effect of an mGluR1 antagonist, which is consistent with the well-documented disruption of mGluR1 signaling by Homer1a. These findings emphasize the important role of mGluR1 in pain-related amygdala plasticity and provide evidence for the involvement of Homer1 proteins in the forebrain in the modulation of pain hypersensitivity.

## Background

Neuroplasticity in the amygdala plays an important role in emotional-affective aspects of pain [1,2]. A growing body of literature is addressing pain-related functions of different amygdala nuclei and signaling mechanisms in these areas [3-16]. Neurotransmission from the lateral amygdala (LA) to the basolateral amygdala (BLA) and further to the central nucleus of the amygdala (CeA) regulates input and output functions of the amygdala. The designation of the latero-capsular division of the central nucleus of the amygdala (CeLC) as the "nociceptive amygdala" emphasizes its role in pain processing and modulation [1,2]. CeLC neurons receive excitatory glutamatergic input directly from neurons in the BLA and inhibitory input via glutamatergic activation of GABAergic neurons in the intercalated cell mass of the amygdala [6].

Group I metabotropic glutamate receptors (mGluR1/5) play an important role in pain-related signaling in the amygdala [3,13-16]. Pain-related neuroplastic changes of excitatory transmission from the BLA to the CeLC are mainly mediated by mGluR1 [6]. Blockade of mGluR1 inhibits arthritis pain-induced audible and ultrasonic vocalizations in rats [15] and decreases excitatory postsynaptic currents (EPSCs) in CeLC neurons in brain slices of arthritis rats [3,6].

Activation of mGluR1/5 leads to the release of intracellular calcium via phospholipase C, which has major cellular consequences such as neuronal excitability changes, enhancement of neurotransmitter release, and potentiation of the activity of NMDA or AMPA receptors [17-20]. Signaling of mGluR1/5 is potently modulated by the family of Homer proteins [21,22]. Homer1 proteins bind to mGluR1/5, and the long splice variants Homer1b and Homer1c, which are constitutively expressed, function as molecular bridges by linking mGluR1/5 to the IP_3 _receptor on the endoplasmatic reticulum [21-23], thereby regulating mGluR-IP_3_R signaling towards the release of calcium from intracellular stores [24]. The short splice variant Homer1a has been identified as an immediate early gene (IEG) following intense neuronal activity [22,25,26]. Expression of Homer1a leads to the disruption of the mGluR-IP_3_R complex [21,23] and to reduced and delayed mGluR-mediated intracellular calcium release [23]. Homer1a has been associated with pain-related plasticity at spinal synapses [27-30] and serves as a endogenous modulator for negative feedback regulation of mGluR-signaling in inflammatory pain conditions [27]. However, pain modulation by Homer1 signaling in the brain is entirely unknown. We explored the contribution of the Homer1a-mGluR signaling complex to pain hypersensitivity and pain-related synaptic plasticity in the amygdala, using Homer1a transgenic mice.

## Findings

This study addressed the interaction of Homer1a and mGluR1 in the amygdala in our kaolin/carrageenan-induced arthritis pain model. We generated mice overexpressing Homer1a in the forebrain and characterized different founder lines [31].

### Generation and maintenance of transgenic mice

The Homer1a-transgenic mouse line was generated, backcrossed to C57BL/6 wild-type strain for more than 10 generations and characterized as described previously [31]. Mice were housed individually in a temperature and 12 h day/night cycle controlled room. All experiments were approved by the Institutional Animal Care and Use Committee (IACUC) at the University of Texas Medical Branch (UTMB) and conform to the guidelines of the International Association for the Study of Pain (IASP) and of the National Institutes of Health (NIH).

### Arthritis pain model

A mono-arthritis was induced in one knee joint as described in detail previously [32]. Briefly, a kaolin suspension (4%, 40 μl) was slowly injected into the joint cavity through the patellar ligament. After repetitive flexions and extensions of the knee for 15 min, a carrageenan solution (2%, 40 μl) was injected into the knee joint cavity, and the leg was flexed and extended for another 5 min. The control group of mice was untreated. We showed previously that intraarticular saline injection does not mimic arthritis-induced changes [3].

### Spinal reflexes

Hindlimb withdrawal reflexes were evoked by mechanical stimulation of the knee joint with increasing intensities, using a forceps equipped with a force transducer system as in our previous studies [5,13,32]. Withdrawal threshold was defined as the minimum stimulus intensity that evoked a withdrawal reflex.

### Vocalizations

Audible (16-20 kHz) and ultrasonic (25 ± 4 kHz) vocalizations were recorded and analyzed as described previously [32,33] using the UltraVox 4-channel system; Noldus Information Technology, Leesburg, VA. Vocalizations were recorded for 1 min before and during application of brief (15 s) innocuous (500 g/30 mm^2^) and noxious (2000 g/30 mm^2^) mechanical stimuli to the knee, using a calibrated forceps [32,33].

### Electrophysiology: patch-clamp recording

Coronal brain slices (300 μm) containing the CeCL were prepared from normal and arthritic mice (6 h postinduction) as described before in rats [3,6,8-11]. Briefly, mice were decapitated and the brains were quickly dissected out and blocked in cold, oxygenated artificial cerebrospinal fluid (ACSF). Whole-cell patch-clamp recordings were obtained from CeLC neurons using the "blind" patch technique as previously described [3,6,8-11]. One neuron was recorded in each slice and 1 or 2 slices were used per animal. 7-(hydroxyimino)cyclopropa[b]chromen-1a-carboxylate ethyl ester (CPCCOEt) was purchased from Tocris Bioscience (Ellisville, MO) and applied by gravity-driven superfusion in the ACSF. The appropriate concentration was determined in our previous study [3].

### Statistical analysis

All data are presented as mean ± SE. For multiple comparisons, Analysis of Variance (ANOVA) was used. Paired Student's t-test was used to compare two sets of data that have Gaussian distribution and similar variances.

### Homer1a expression in H1a-mice

**Figure 1:**
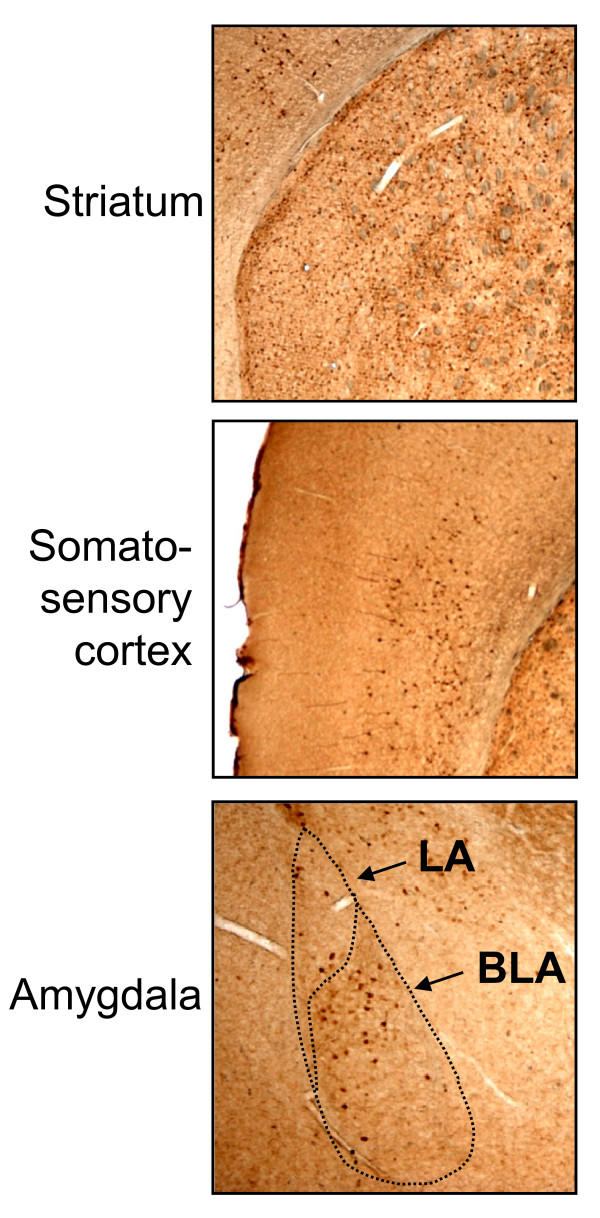
**Characterization of transgenic mice expressing Homer1a in the forebrain**. Immunohistochemical characterization of myc-tagged Homer1a in forebrain sections of transgenic mice overexpressing Homer1a (H1a-mice) using an anti-myc antibody. Anti-myc antibody detected Homer1a expression in the striatum, in the somatosensory cortex and in the amygdala (BLA and LA).

Immunohistochemistry was used to detect the expression of the myc-tagged Homer1a protein. Here we used a transgenic line overexpressing Homer1a uniformly strong in the striatum, in layers V and VI of the somatosensory cortex, and in the amygdala (Figure1). In the amygdala, Homer1a was mainly expressed in the BLA and to a lesser extend in the lateral amygdala (LA; Figure1). We used this myc-tagged Homer1a-overexpressing mouse-line ("H1a-mice") to address the significance of the mGluR1 signaling in amygdala neurons in arthritis pain. Non-Homer1a expressing littermates were used as control mice ("wild type").

### Pain behavior in H1a-mice

**Figure 2:**
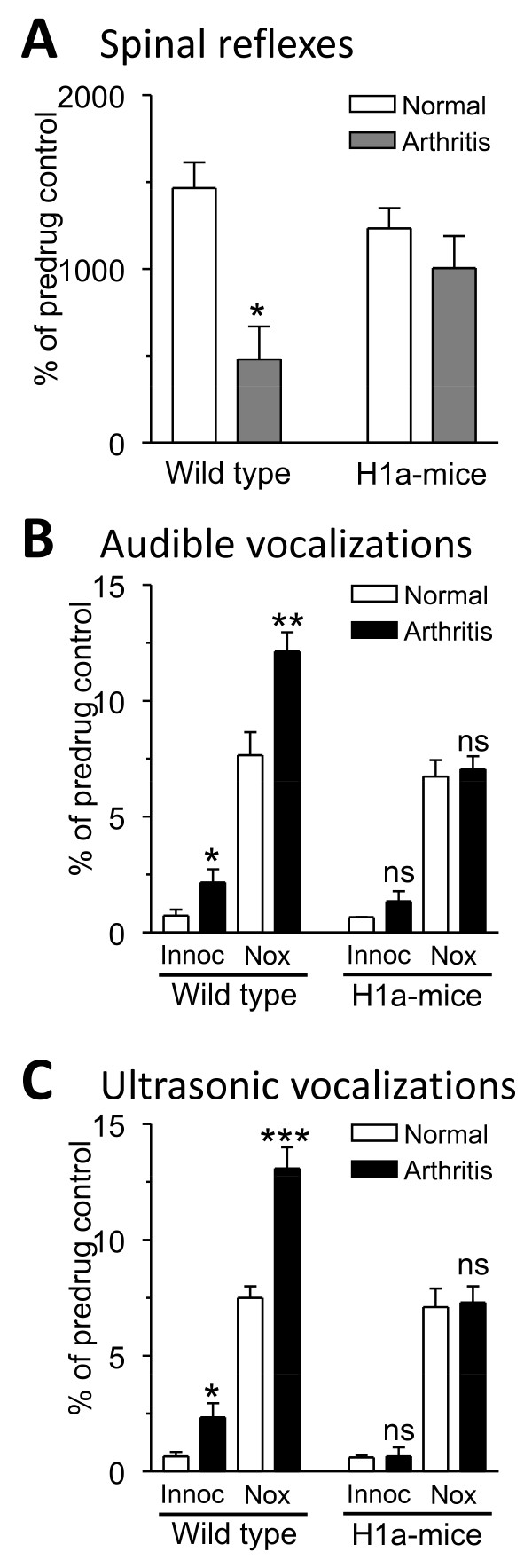
**Pain behaviors in wild type and transgenic mice**. **(A) **Hindlimb withdrawal thresholds decreased in wild-type mice 6 h postinduction of arthritis (n = 3), but not in mice overexpressing Homer1a (H1a-mice; n = 3). **(B, C) **Audible and ultrasonic vocalizations increased in wild-type mice 6 h postinduction of arthritis (n = 6), but not in H1a-mice (n = 6). *,**,*** P < 0.05, 0.01, 0.001 (compared to normal pre-arthritis values in the same animal; paired t-test).

The knee joint arthritis led to the development of increased pain responses in wild-type mice, which is reflected in the decrease of hindlimb withdrawal thresholds (n = 3; Figure2A) and an increase in the duration of audible and ultrasonic vocalizations (n = 6; Figures2Band2C) measured 6 h postinduction. In contrast, H1a-mice did not develop mechanical hypersensitivity (n = 3; Figure2A) and showed no change in vocalizations (n = 6; Figures2Band2C), suggesting that the expression of Homer1a protects against arthritis pain.

### Synaptic transmission in the amygdala in H1a-mice

**Figure 3:**
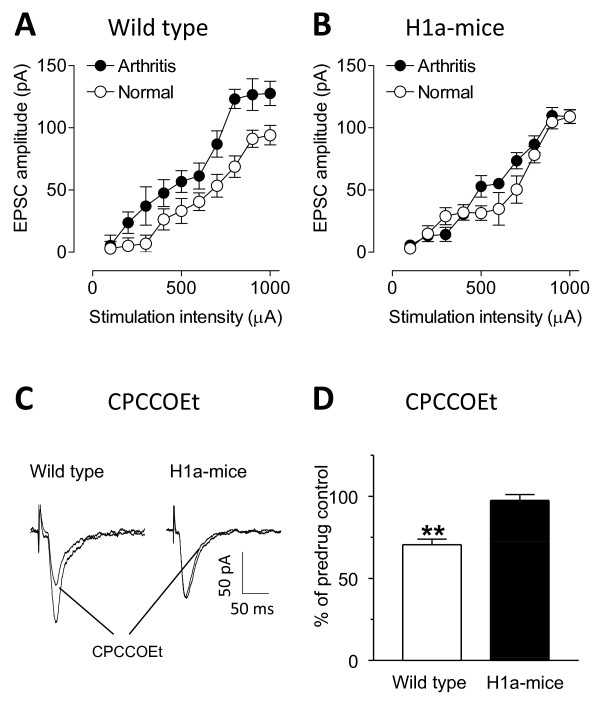
**Synaptic transmission in the amygdala in brain slices from wild type and transgenic mice**. Whole-cell patch recordings of excitatory transmission at the BLA-CeLC synapse in brain slices from wild-type and Homer1a overexpressing mice (H1a-mice) with and without arthritis (6 h postinduction). **(A) **Input-output functions of monosynaptic EPSCs in CeLC neurons increased in wild-type mice with arthritis (n = 5 neurons) compared to mice without arthritis (normal; n = 4) **(B) **Input-output functions of monosynaptic EPSCs in CeLC neurons were not different in brain slices from H1a-mice with arthritis (n = 5 neurons) and without arthritis (n = 4). **(C) **Monosynaptic EPSCs recorded in an individual CeLC neuron in a brain slice from an arthritic wild-type mouse and in another CeLC neuron from an arthritic H1a-mouse before and during CPCCOEt (10 μM; average of 8-10 traces). **(D) **CPCCOEt (10 μM) inhibited EPSCs in slices from arthritic wild-type mice (n = 5 neurons) but not in slices from arthritic H1a-mice (n = 5 neurons). Bar histograms show normalized drug effects (expressed as percent of predrug control, set to 100%). ** P < 0.01 (compared to predrug control in the same neurons; paired t-test).

We went on to investigate the effect of Homer1a overexpression on pain-related changes of excitatory synaptic transmission and mGluR1 function in the CeLC. Whole-cell patch-clamp technique was used to record monosynaptic EPSCs evoked in CeLC neurons by electrical stimulation in the BLA in brain slices from wild type and from H1a-mice, with or without arthritis (6 h postinduction, Figure3). In agreement with previous studies in rats [3,6,8-11], input-output functions of excitatory transmission increased in CeLC neurons in wild-type mice with arthritis (n = 5 neurons) compared to mice without arthritis (n = 4) significantly (P < 0.0001, F_1,70 _= 39.53; Figure3A). Blockade of mGluR1 with a selective antagonist (CPCCOEt, 10 μM, n = 5 neurons) inhibited excitatory synaptic transmission significantly in wild-type mice with arthritis (P < 0.01, paired t-test, compared to predrug; Figures3Cand3D). Our previous studies showed that this effect was presynaptic [3,6]. In H1a-mice, baseline excitatory synaptic transmission (no arthritis, n = 4 neurons; Figure3B) was not significantly different from that in wild-type mice (P > 0.05, F_1,60 _= 3.63; Figure3A). Different than in wild-type mice, however, excitatory synaptic transmission in H1a-mice did not change in arthritis (n = 5 neurons, P > 0.05, F_1,70 _= 3.86; Figure3B). Importantly, CPCCOEt (10 μM) had no effect on excitatory transmission in slices from H1a-mice with arthritis (n = 5 neurons; Figures3Cand3D).

Our findings confirm previous studies performed in rats where mGluR1 has been shown to play an important role in pain-related synaptic plasticity in the CeLC [3,6]. The novel result of the present study is that interactions of mGluR1 with the scaffolding proteins of the Homer1 family in the forebrain play an important role in inflammatory pain hypersensitivity as well as in synaptic plasticity in the amygdala. Homer1a serves here as an endogenous "antagonist" of group I mGluR signaling as proposed previously in the spinal cord [22,27,34] and prevents pain-related behavioral and synaptic changes. As in our previous study in rat brain slices [3] the mGluR1 antagonist CPCCOEt had no effect on synaptic transmission under normal conditions but strongly inhibited synaptic transmission in arthritis rats. However, this effect was lost in mice overexpressing Homer1a, which can be explained by Homer1a disrupting mGluR1 signaling hence occluding the inhibitory effect of the mGluR1 antagonist seen in wild-type mice. The disruptive effect of Homer1a overexpression in the BLA rather than CeA is consistent with a presynaptic site of action previously shown for mGluR1 at the BLA-CeA synapse [3,6].

## Conclusions

Our findings emphasize the importance of mGluR1-Homer1 interactions in amygdala neurons in pain and provide evidence for a protective role of the activity-induced regulator, Homer1a, in the forebrain in inflammatory pain. It is conceivable that expression of Homer1a in forebrain areas other than the amygdala may also disrupt pain-related synaptic plasticity and prevent the development of pain hypersensitivity. Candidate brain areas include the anterior cingulate cortex and insular cortex. Disrupting pain-related plasticity in the anterior cingulate cortex has recently been shown to alleviate neuropathic pain [35].

## List of abbreviations

ACSF: artificial cerebrospinal fluid; BLA: basolateral nucleus of the amygdala; CeA: central nucleus of the amygdala; CeLC: latero-capsular division of the CeA; CPCCOEt: 7-(hydroxyimino)cyclopropa[b]chromen-1a-carboxylate ethyl ester; EPSC: excitatory postsynaptic current; H1a: Homer1a; LA: lateral nucleus of the amygdala; mGluR: metabotropic glutamate receptor.

## Competing interests

The authors declare that they have no competing interests.

## Authors' contributions

AT, generated the transgenic mice, performed histological analysis of Homer1a expression in the brain, and wrote the first draft of the manuscript. YF performed patch-clamp recordings and behavioral tests, analyzed data, and provided figures. RK supervised the transgenic experiments data analysis and RK and VN conceptualized the hypothesis. VN designed and supervised the electrophysiological and behavioral experiments and finalized the manuscript. All authors read and approved the manuscript.
